# Transcriptome sequencing of Atlantic salmon (*Salmo salar* L.) notochord prior to development of the vertebrae provides clues to regulation of positional fate, chordoblast lineage and mineralisation

**DOI:** 10.1186/1471-2164-15-141

**Published:** 2014-02-19

**Authors:** Shou Wang, Tomasz Furmanek, Harald Kryvi, Christel Krossøy, Geir K Totland, Sindre Grotmol, Anna Wargelius

**Affiliations:** 1 Department of Biology, University of Bergen, Bergen, Norway; 2 Institute of Marine Research, Bergen, Norway

**Keywords:** Atlantic salmon, Notochord, RNA-seq, *col11a2*, Hox

## Abstract

**Background:**

In teleosts such as Atlantic salmon (*Salmo salar* L.), segmentation and subsequent mineralisation of the notochord during embryonic stages are essential for normal vertebrae formation. However, the molecular mechanisms leading to segmentation and mineralisation of the notochord are poorly understood. The aim of this study was to identify genes/pathways acting in gradients over time and along the anterior-posterior axis during notochord segmentation and immediately prior to mineralisation of the vertebral bodies in Atlantic salmon.

**Results:**

Notochord samples were collected from unsegmented, pre-segmented and segmented developmental stages. In each stage, the cellular core of the notochord was cut into three pieces along the longitudinal axis (anterior, mid, posterior). RNA was sequenced (22 million pair-end 100 bp/ library) and mapped to the salmon genome. 66569 transcripts were predicted and 55775 were annotated. In order to identify possible gradients leading to segmentation of the notochord, all 71 notochord-expressed hox genes were investigated, most of them displaying a typical anterior-posterior expression pattern along the notochord axis. The clustering of hox genes revealed a pattern that could be related to notochord segmentation. We further investigated how mineralisation is initiated in the notochord, and several factors related to chondrogenic lineage were identified (s*ox9*, *sox5*, *sox6, tgfb3, ihhb* and *col2a1*), suggesting a cartilage-like character of the notochord. KEGG analysis of differentially expressed genes between stages revealed down-regulation of pathways associated with ECM, cell division, metabolism and development at onset of notochord segmentation. This implies that inhibitory signals produce segmentation of the notochord. One such potential inhibitory signal was identified, *col11a2*, which was detected in segments of non-mineralising notochord.

**Conclusions:**

An incomplete salmon genome was successfully used to analyse RNA-seq data from the cellular core of the Atlantic salmon notochord. In transcriptome we found; hox gene patterns possibly linked to segmentation; down-regulation of pathways in the notochord at onset of segmentation; segmented expression of *col11a2* in non-mineralised segments of the notochord; and a chondroblast-like footprint in the notochord.

## Background

The notochord is a midline structure that appears early in the embryo of all vertebrates, and has several important functions. It provides internal hydroskeletal support, until this role is taken over by the vertebral column. It also produces secreted factors during development that provide position and fate signals to adjacent ectoderm, paraxial mesoderm and endoderm along the dorso-ventral axis [1-5]. In recent years, additional functions of the notochord have been unravelled. Segmentation and subsequent mineralisation of the notochord comprise the initial morphogenic process in formation of the vertebral column in Atlantic salmon (*Salmo salar* L.) [6] and in zebrafish (*Danio rerio*) [2,7]. Segmented mineralisation of the notochord determines the localization of vertebral bodies, and notochord mineralisation is thereby crucial for normal formation of the vertebrae, as the functional study by Willems et al. [8] has demonstrated. Furthermore, pathological processes that disrupt notochord mineralisation may lead to malformation of vertebrae [9]. However, mechanisms initiating segmentation and mineralisation within the notochord are currently unknown, and further studies of this topic are needed.

The salmon embryo provides a good model for studies of development and differentiation, due to its slow early development and large size, allowing pure samples of the cellular core of the notochord to be isolated. This has facilitated some detailed molecular studies of salmon notochord, and the discovery of notochord-specific molecules such as *vimentin* and *elastin*[10]. Moreover, the development of the notochord and the vertebral column of Atlantic salmon embryos and larvae have previously been the subjects of detailed morphological studies [6,9,11-13]. This has made it possible to characterize in detail both notochord segmentation and the subsequent mineralisation that nucleates vertebral development.

As development of the salmon notochord proceeds, segmentation is initially observed in the notochord epithelium as metameric bands of cells with alternating cell-axis orientations [6] concurrent with expression of alkaline phosphatase (ALP) in every second band [12], both processes defining the future positions of vertebral and intervertebral segments. In the following stage, possibly in order to permit further growth of the vertebrae, sclerotome-derived osteoblasts differentiate on the surface of the notochord centrae to form bone [9,11].

Despite the importance of the notochord in the initial shaping of the vertebral column, the genes involved and the network of transcriptional regulators controlling them are still incompletely characterized. An overall transcriptome assessment using RNA-seq on critical stages during the early segmentation stage of salmon notochord might reveal pathways associated with the segmentation and subsequent mineralisation processes. Possible factors associated with segmentation of the notochord could be members of the hox gene family, as these molecules are known to confer patterning formation along the anterior-posterior body axis in all vertebrates. In zebrafish, four hox genes have been found to be differentially expressed along the anterior-posterior axis of the notochord [14], which indicates that these genes may play a role in metameric morphogenic processes within the notochord of teleosts. In Atlantic salmon, 118 hox genes have been identified, including 8 pseudogenes [15], but none of them have been studied with regard to notochord development. Furthermore, the differentiation of chordocytes, and specific features of notochord mineralisation, might posibly be elucidated via a transcriptome study, as factors typical of chondrogenesis and osteogenesis could be identified. For instance, both the hedgehog and Wnt signalling pathways play important roles in formation of the notochord sheath and the patterning of the vertebral column during early development in the mouse [16].

The primary objective of the present study was to relate the transcriptome of the notochord to morphological events in un-segmented notochord (510 day), pre-segmented notochord (610 day) and segmented notochord to the ongoing mineralisation of the sheath (710 day). This study employed high-throughput RNA sequencing technology on dissected anterior-posterior notochord segments to explore, at global level, the pathways and genes expressed in the notochord that might contribute to notochord development, segmentation and subsequent mineralisation in salmon and related species.

## Methods

### Rearing of embryos and notochord dissection

**Figure 1:**
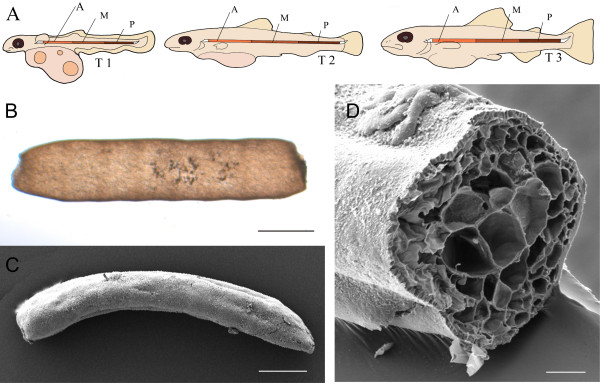
**Salmon notochord sampling scheme and morphology. (A)** Notochord sampling at three developmental stages calculated in day degrees (d°) (T1:510 ± 20 d°, T2: 610 ± 20 d°, T3: 710 ± 20 d°), and further division into segments (A: anterior, M: mid, P: posterior) along the longitudinal axis. **(B)** A freshly dissected notochord sample, from stage T3. Stereoscope photo, scale bar: 500 um. **(C, D)**: Scanning electron micrographs of dissected notochord. Notice the smooth, sheath-free surface and the large central chordocytes and the epithelium germ layer (chordoblasts) in transverse section **(D)**. Scale bar: 400 um in **(C)** and 100 um in **(D)**.

Salmon embryos were collected from a local hatchery (Marine Harvest, Alvøen, Norway). Yolk-sac embryos were kept in a refrigerator at around 4°C during the sampling period. Developmental stages were classified by day degrees (day), which are defined as the sum of the daily mean ambient water temperatures (˚C) for each day of development. Based on our earlier morphologic observations [6], three development stages were selected spanning from the un-segmented notochord, through the segmental differentiation of notochord epithelium, to the pre-mineralisation stage of the notochordal sheath (Figure 1A): 510 ± 20 d° (T1:un-segmented notochord); 610 ± 20 d° (T2:pre-segmented notochord); 710 ± 20 d° (T3: segmented notochord/mineralisation in the sheath). Pure fractions of notochord cells were detached from the fibrous sheath, following the procedure described by Sagstad et al. [10]. At each stage, the notochord was dissected into three pieces along the longitudinal axis (A: anterior, M: mid, P: posterior) (Figure 1A), before being fresh-frozen in liquid nitrogen and stored at −80°C. The notochord samples were intact with traces neither of the sheath nor of surrounding tissues (Figure 1B,C).

### Histology

Samples of notochord with surrounding tissues for light microscopy, and pure notochord fractions for scanning electron microscopy (SEM), were fixed and processed as described by Grotmol et al. [6]. Histological slides were photographed in an Olympus Vanox X3 microscope (Olympus, Tokyo, Japan), while samples for SEM were studied in a Zeiss FESEM Supra 50 field-emission scanning EM (Oberkochen, Germany).

### Total RNA extraction and transcriptome sequencing

Total RNA was extracted from notochord pieces using Rneasy Mini Kit (Qiagen, Hilden, Germany), according to the manufacturer’s instruction. Only RNA samples with A260/280 within 1.9-2.1 and A260/230 > 1.8 measured by Nanodrop (ND-1000, Thermo Scientific, Wilmington, USA), were used for pooling. In total, notochord pieces (anterior, mid-, posterior parts in separate tubes) from 36 fish from T1, 18 fish from T2 and 27 fish from T3 were pooled and prepared for nine total RNA libraries (at least 6 μg RNA/library, labeled as: T1A, T1M, T1P, T2A, T2M, T2P, T3A, T3M, T3P). RNA integrity was checked for each library (RIN value > 9, except for T3M) by Agilent 2100 Bioanalyzer (RIN values for individual library were listed in Additional file 1: Table S1). RNA libraries were prepared by Fasteris Life Science (Geneva, Switzerland). Briefly cDNA libraries were prepared using random hexamer primers and samples were sequenced using 2x100bp paired-end high-throughput mRNA sequencing (RNA-seq) in single lane on Illumina Hiseq 2000 (Illumina, San Diego, CA). Due to the time-consuming dissecting procedures involved and limited tissue mass, single libraries without replicates were sequenced.

### Bioinformatical analysis of RNA-seq

**Figure 2:**
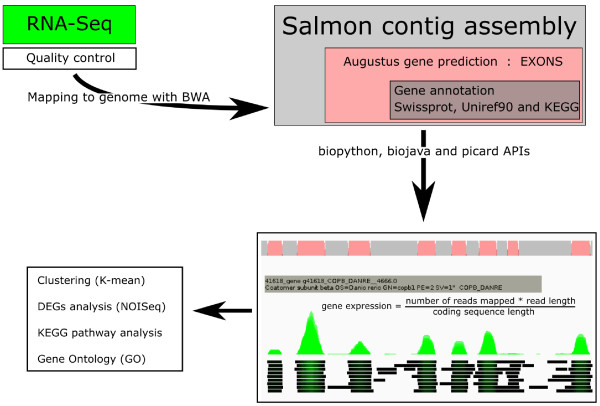
**Bioinformatics analysis pipeline for RNA-seq.** Short reads were checked for quality score and nucleotide distribution, and reads were not trimmed. Reads were mapped to the genome with BWA. Predictions of coding sequences (exon) of the salmon contig assembly were performed using Augustus algorithm. Gene annotations were done by homologous sequence inference against Swissprot, Uniref90 and KEGG databases. Total mapped reads to predicted exons were summarized and normalized using the longest transcript length among all libraries and total number of reads per library. 66569 transcripts/genes were identified in the raw gene list with more than three reads per gene. Differentially expressed gene (DEG) analysis was performed using NOISeq. DEG’s were further clustered by k-mean clustering, KEGG pathway and Gene Ontology ID.

The workflow of the bioinformatics analysis is summarised in Figure 2. Further, The raw data has been deposited and can be found at The Sequence Read Archive (SRA) at NCBI (Accession ID: SRA129427). Library quality control was performed by a titration run with 50 bp reads on HiSeq, followed by a manual blast of 50 fasta sequences against salmon EST databases and quality check of 100,000 fastq reads for each nucleotide position from each library (Additional file 2: Figure S1A). Fewer than 10% fasta sequences were annotated as ribosomal genes, and more than 60% aligned to salmonids ESTs (data not shown). The distributions of nucleotides in all reads were checked, although the first 13 bp in all reads are biased, probably due to random hexamer priming in the Illumina cDNA library preparation protocol (Additional file 2: Figure S1B), as previously reported [17]. To obtain gene expression values of each gene, sequences were mapped against a draft salmon genome contig assembly (Acc. No. AGKD00000000.1). To acquire gene prediction for the draft salmon genome, Augustus gene prediction software [18] was trained using PASA gene candidates [19]. The PASA gene candidates were obtained by mapping salmon ESTs to the draft salmon genome contig assembly. The Augustus gene software only predicted coding sequences without UTRs. Predicted genes were annotated with various resources, including Swissprot, Uniref90 and KEGG. RNA-seq reads of 100 bp were aligned to the genome assembly with the Burrows-Wheeler Alignment tool (BWA) [20] in paired-end mode. Since the genome reference was fragmented, the paired-end information was only checked in cases where both reads in a pair mapped to the same contig. Aligned reads were analyzed with biopython, biojava and picard APIs (application programming interface).

The commented java source code based on the Picard API for extracting the read count per Augustus predicted gene is available at https://marineseq.imr.no/salmon/annot2013/. “In silico” gene expression was calculated as: (number of mapped reads*read length)/ (predicted coding sequence length). In cases where the mapped reads overlapped coding sequence and the UTR, only the part of the read that mapped to the coding sequence was counted. If a read mapped to a coding sequence with a 90% match, only 0.9 was added. Reads with an identity match to the genome below 90% were removed from the analysis. Normalization of expression values among libraries was completed by counting the total number of reads from each library and then recalculating, so that each library was given the same number of counts.

### Differential gene expression

Differentially expressed genes (DEG) were selected by using NOISeq [21] with a threshold 0.8. Since we did not have true biological replicates, we either pooled three segments (A, M, P) of each developmental stages (labelled as sumT1, sumT2, sumT3), or three stages (T1, T2, T3) for each segment (sumA, sumM, sumP). We ran NOISeq for both spatial and temporal comparison on pooled libraries, using library-normalized raw read count for each gene. K-mean clustering was used to group genes with similar patterns of expression. Gene Ontology [22] was used to pick up genes that were annotated with ECM. The output ECM gene list could be cross-checked for DEGs from NOISeq. KEGG pathways analysis [23] was performed by mapping the KEGG annotated DEGs from NOISeq to KEGG pathways as described in the KEGG Mapper tool. Both raw expressions of genes and DEGs as fold change were plotted in pathways, and the ratio of number of up-regulated genes/number of down-regulated genes, or vice versa, were calculated as a means of ranking up-/down-regulated pathways.

### Quantitative real-time PCR

Quantitative real-time PCR (qPCR) was performed using SYBR Green PCR Master Mix (Applied Biosystems Inc., Foster City, CA, USA). Total RNA of all libraries was extracted from dissected notochord, following the same procedure as described for transcriptome sequencing from another batch of salmon embryos [10]. Only RNA samples with A260/280: 1.8-2.0 were pooled from two additional batches of salmon embryos, and they were used for cDNA synthesis with SuperScript VILO cDNA Synthesis Kit (Invitrogen), according to the manufacturer’s guidelines. After TURBO DNA-free kit (Ambion) treatment and sodium acetate precipitation, qPCR reactions were run in a 7900HT Fast Real-Time PCR System (Applied Biosystems Inc.). The conditions for all reactions were 50°C for 2 min, followed by 95°C for 10 min, 40 cycles of 95°C for 15 sec, followed by 60°C for 1 min. Genomic fragments from the draft salmon genome assembly (Acc. No. AGKD00000000.1) were used to design primers and probes, as shown in Additional file 3: Table S2. Primers were designed using Primer Express v2.0 software (Applied Biosystems Inc.) and Primer-BLAST [24]. *Elongation factor 1* α (*ef1a*) was used as reference gene for its stable expression in salmon tissues [25], including notochord [10,13,26]. *ef1a* was expressed at the same level in all libraries (Gene expression data found at https://marineseq.imr.no/salmon/annot2013/). Three biological replicate cDNAs were made for gene expression analysis. Standard curve analysis was performed to confirm similar amplification efficiency in target genes and the reference gene with a validation step. A no-template-control (NTC) and an RNA sample without reverse transcription (−RT), were used to control for contamination of external and/or genomic DNA in reactions. Melting curve analyses were performed for each primer pair in order to confirm unique amplicon reaction. One-way ANOVA (p < 0.05) and Tukey’s multiple comparison, were performed in GraphPad Prism v5 (GraphPad Software, San Diego, CA. USA) to detect DEG in summed libraries (sumA-P and sumT1-T3) following analysis of the qPCR data.

### In situ hybridization

In situ hybridization was performed according to Krossøy et al. [27]. Primer sequences for *col11a2* are listed in Additional file 3: Table S2.

## Results

### Transcriptome features, annotation and quality

**Figure 3:**
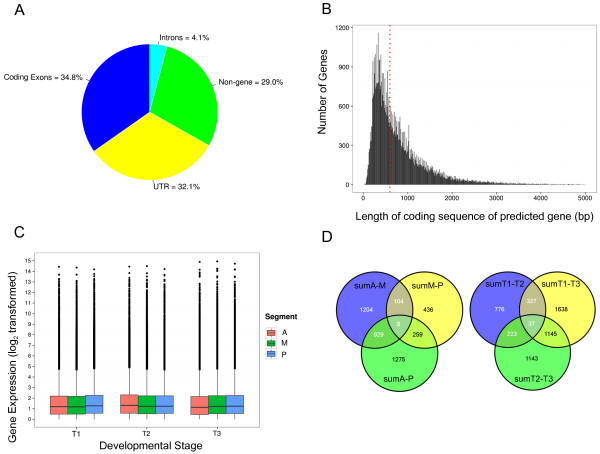
**Overview of the salmon notochord transcriptome from RNA-seq. (A)** Genomic location of mapped reads in percentage in all salmon notochord libraries. **(B)** Distribution of Augustus-predicted gene length in basepair (bp) < 5000 bp; the others were not plotted. The red dotted line indicates the average gene length of all genes, which was 872 bp. **(C)** Distribution of expression (in log2 scale) of all genes in libraries, grouped by development stages (T1:510 ± 20 d°, T2: 610 ± 20 d°, T3: 710 ± 20 d°). **(D)** Venn diagrams showing number of differentially expressed genes from NOISeq between pair-wise comparison between summed spatial libraries (SumA, SumM, SumP) and summed temporal libraries (SumT1, SumT2, SumT3). Abbreviations: A (anterior), M (mid), P (posterior).

Around 22 million 100 bp paired-end reads were collected from each library, out of which 70% of the reads were mapped to the draft salmon genome (Additional file 1: Table S1). Similar mapping percentages were achieved using another mapping tool, Bowtie2 [28] (data not shown). We used at least three reads mapped/Augustus-predicted genes to output 66569 genes as the raw gene list, of which, 55775 genes were annotated using the Uniprot/Swissprot database. The whole gene set was additionally annotated with Uniref90 and KEGG. The Augustus gene prediction, predicted gene sequences and annotation files can be downloaded from https://marineseq.imr.no/salmon/annot2013/. Out of all the mapped reads, 34.8% mapped to predicted coding exons in the draft salmon genome; 32.1% mapped to regions up to 1000 bp downstream of predicted genes, and thus estimated the expression of untranslated regions (3′ UTRs); 29% and 4.1% mapped to non-genic regions and intron regions, respectively (Figure 3A). The average predicted coding sequence length of predicted gene fragments was around 872 bp (Figure 3B). As a measure of the quality of all libraries, expression distribution was measured in each library, which were very similar throughout, with an average expression level of about 1 (Figure 3C). Pair-wise comparisons of the number of DEGs between summed notochord libraries (either spatially or temporally) using NOISeq are shown in Figure 3D. From sumA-P comparison, 2470 DEGs were identified (1568 annotated and 902 unknown genes), of which 1263 genes were up-regulated and 1207 were down-regulated during the sampling period. From sumT1-T3 comparison, 3147 DEGs were identified (2655 annotated and 483 unknown genes), of which 1531 genes were up-regulated and 1616 down-regulated.

### qPCR validation of RNA-seq gene expression values

**Figure 4:**
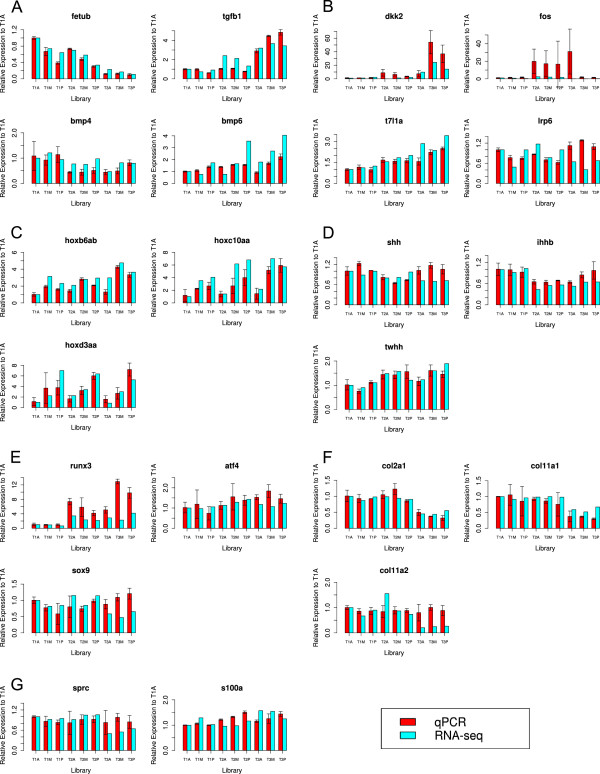
**Validation of RNA-seq by qPCR.** RNA-seq expression of 22 selected genes was confirmed by independent quantitative RT-PCR (qPCR) method. Expression pattern of four genes (*fetub*, *tgfb1*, *bmp4*, *bmp6*) involved in transforming growth factor-beta (TGFβ)/bone morphogenic protein (BMP) signaling pathways **(A)**; four genes (*dkk2*, *fos*, *t7l1a*, *lrp6*) in Wnt signaling pathway **(B)**; three hox genes (*hoxb6ab*, *hox10aa*, *hoxd3aa*) **(C)**; 3 Hedgehog genes (*shh*, *ihhb*, *twhh*) **(D)**; three transcriptional factors (*runx3*, *atf4*, *sox9*) involved in osteochondro-progenitors lineage **(E)**; three fibrillar collagen genes (col2a1, col11a1, col11a2) **(F)**; two calcium binding proteins (sprc, s100a) **(G)** across all nine notochord libraries (T1A, T1M, T1P, T2A, T2M, T2P, T3A, T3M, T3P) at three developmental stages (T1:510 ± 20 d°, T2: 610 ± 20 d°, T3: 710 ± 20 d°). Of the expression of individual genes in each panel, the X-axis indicates notochord libraries; the Y-axis indicates the gene expression in one library as fold change relative to T1A library (T1A: anterior part of salmon notochord at 510 ± 20 d° stage), measured with RNA-seq (cyan) and qPCR (red). Error bar indicates mean ± SD. Overall, Abbreviations: A (anterior), M (mid), P (posterior). Gene name: Fetuin-B (*fetub*); Transforming growth factor beta-1 (*tgfb1*); Bone morphogenetic protein 4 (*bmp4*); Bone morphogenetic protein 6 (*bmp6*); Low-density lipoprotein receptor-related protein 6 (*lrp6*); Dickkopf-related protein 2 (*dkk2*); Transcription factor 7-like 1-A (*t7l1a*); Proto-oncogene c-Fos (*fos*); Homeobox protein HoxB6ab (*hoxb6ab*); Homeobox protein HoxC10aa (*hoxc10aa*); Homeobox protein HoxD3aa (*hoxd3aa*); Indian hedgehog B protein (*ihhb*); Sonic hedgehog protein A (*shh*); Tiggy-winkle hedgehog protein (*twhh*); Transcription factor SOX-9 (*sox9*); Runt-related transcription factor 3 (*runx3*); Cyclic AMP-dependent transcription factor ATF-4 (*atf4*); Protein S100-A1 (*s100a*); SPARC(*sprc*); Collagen alpha-1(II) chain (*col2a1*); Collagen alpha-1(XI) chain (*col11a1*); Collagen alpha-2(XI) chain (*col11a2*).

A total of 23 genes were selected for validation of RNA-seq gene expression by independent quantitative RT-PCR. Of these, all except *runx2* were expressed (Ct < 30 cycles or deltaCt <8 between the target gene and the reference gene *Elongation factor 1*α). A high correlation was detected between RNA-seq and qPCR gene expression level as relative fold change to T1A notochord library. In general, most of the genes showed the same trend in both methods (Figure 4). The expression of four genes (*fetub*, *tgfb1*, *bmp4*, *bmp6*), involved in the TGFβ/BMP signaling pathways (Figure 4A); four genes (*lrp6*, *dkk2*, *t7l1a*, *fos*) involved in the Wnt signalling pathway (Figure 4B); three hox genes (*hoxb6ab*, *hoxc10aa*, *hoxd3aa*) (Figure 4C); three Hedgehog genes (*ihhb*, *shh*, *twhh*) (Figure 4D); three major transcriptional factors (*sox9*, *runx3*, *atf4*) in osteogenic and chondrogenic lineages of mesenchyme stem cells (Figure 4E); three genes (*col2a1*, *col11a1*, *col11a2*) coding fibrillar collagen chains (Figure 4F) and two calcium-binding protein-coding genes (*s100a*, *sprc*) (Figure 4G) were shown. Further statistical analysis (NOISeq and one-way ANOVA among summed libraries (sumA-P or sumT1-T3) indicated that 13 of 22 genes were differentially expressed in at least one comparison between summed libraries. Results from the statistical analysis of differential gene expression in both methods are shown in Additional file 3: Table S2. Twelve of 13 DEGs were confirmed by qPCR assay (except *fos*, which showed large variation between libraries). Among the DEGs confirmed with both methods, four DEGs (*bmp6*, *hoxb6ab*, *hoxc10aa*, *hoxd3aa*) were significantly up-regulated from anterior to posterior (sumA-P) based on RNA-seq data. Four DEGs (*tgfb1*, *bmp6*, *dkk2*, *runx3*) were significantly up-regulated from T1 to T3, while four DEGs (*fetub*, *bmp4*, *col2a1*, *col11a1*) were significantly down- regulated from T1 to T3.

### Expression of Hox genes within the notochord

**Figure 5:**
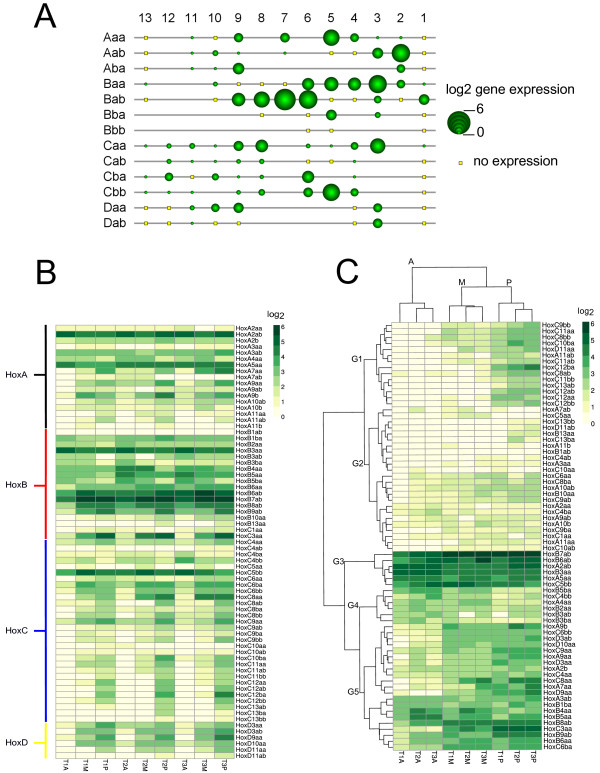
**Hox gene expression from RNA-seq within the salmon notochord during segmentation.** 71 out of 118 salmon hox genes were expressed and the average expression (on log2 scale) of hox genes in all salmon notochord libraries **(A)**. **(B)** Heatmap showing RNA-seq expression (in log2 scale) of hox genes in nine notochord libraries; x-axis primarily grouped by developmental stage (T1:510 ± 20 d°, T2: 610 ± 20 d°, T3: 710 ± 20 d°); y-axis grouped by four major clades in hox clusters. **(C)** K-mean clustered heatmap showing RNA-seq expression (in log2 scale) of hox genes in nine notochord libraries. Lowly expressed posterior genes were represented in G1; generally lowly expressed genes were grouped in G2; most highly-expressed genes were grouped in G3; G4 represented anteriorly-expressed genes and G5 shown the highly-expressed hox genes either in mid- and posterior segments or had no clear pattern. The horizontal clustering indicates a closer distance in overall expression pattern of hox genes between temporal libraries (T1, T2, T3) and, to a further distance between spatial libraries (A, M, P). Abbreviations: A (anterior), M (mid), P (posterior). Note that a total of 110 hox genes were plotted in all figures **(A-C)**, since the rest 8 hox genes are pseudogenes.

Fasta sequences of hox genes were further blasted against the NCBI nucleotide database, and annotated with full-length salmon hox genes with accession number provided in [15]. The expression of genes with the same annotation was fused before log transformation. In this study, 71 out of 118 hox genes in 13 clusters were expressed in the notochord (Figure 5A). Most of Hox clusters displayed the expected collinear expression, with some, such as members of the Hox A and B clusters, having the highest expression in the anterior samples, while others had the opposite pattern of expression, many of which were found in the C and D clusters (Figure 5A-C). The cluster with highest gene expression activity was the B cluster, while less active genes were found in A, C and D clusters (Figure 5A). Clustering of all hox genes revealed that about half of the 71 display a very low expression profile (G1 and G2 nodes in Figure 5C). Interestingly, several members of classical anterior clusters (A and B) contain many hox genes which have a posterior expression pattern (G5 node, in Figure 4C). Only a few genes had the highest expression in the anterior part of the notochord, including; *hoxb5ba*, *hoxc4bb*, *hoxb4bb*, *hoxa4aa*, *hoxb2aa*, *hoxb3ab* and *hoxb3ba* (G4 node, in Figure 5C). Also, in the whole dataset, there seems to be an overrepresentation of hox genes having a posterior expression pattern (Figure 4C). For most of the genes, expression was consistent throughout all three developmental stages. However, in the highly expressed hox genes (G5 node, in Figure 4C), gene expression increased at 610 dº. The posterior expression of a 3′ located hox gene (*hoxd3aa*), *hoxb6ab* and another 5′ located hox gene (*hoxc10aa*), were validated by qPCR (Figure 4C).

### Expression of key molecules in chondrogenic lineage, ECM formation and mineralisation

**Figure 6:**
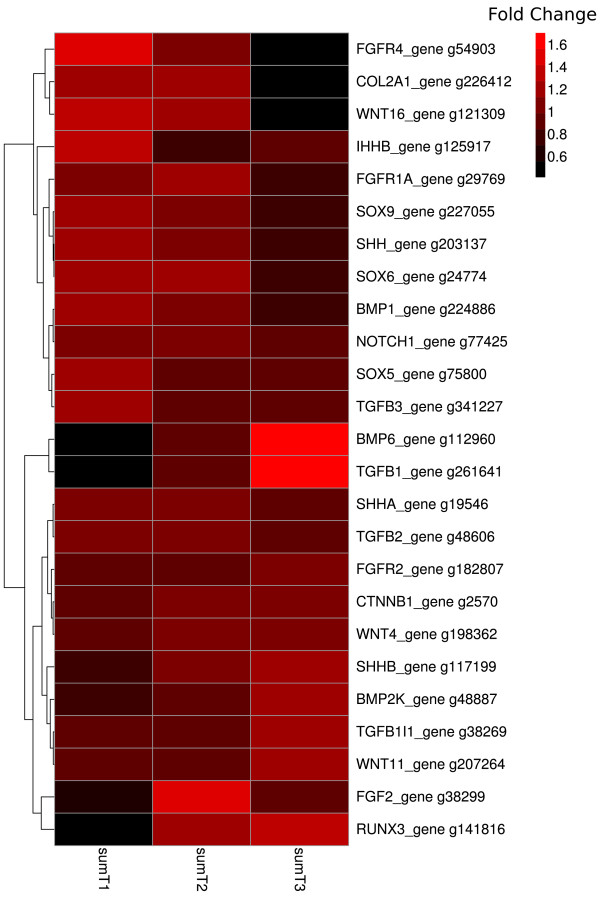
**Clustered heatmap of chondrogenic factors expressed in the notochord.** Gene networks that possibly direct chondrogenic differentiation pathway from mesenchyme stem cells were expressed in the notochord RNA-seq dataset. Genes were filtered with a threshold of blast score > 300 and represented by genes with highest blast score if they had the same gene symbol. Gene expression from RNA-seq from three spatial segments were summed and represented as sumT1, sumT2 and sumT3 (T1:510 ± 20 d°, T2: 610 ± 20 d°, T3: 710 ± 20 d°) and plotted as fold change to the average among the summed libraries. Genes were clustered according to their expression pattern.

To determine if there exist common pathways that determine the differentiation of osteoblast, chondroblast and chordoblast (notochord epithelium cells), and thus a possible evolutionary link in tissue morphogenesis, key genes directing mesenchyme stem cells toward osteogenic and chondrogenic lineages were searched, and the expressed genes are listed in Figure 6. *Runx2* (*cbfa1*), *osterix* and *osteocalcin* (*bgp*), which are genes known to be involved in osteogenesis, were not detected in our analysis, and no expression of *runx2* was confirmed by qPCR. Interestingly, *runx1* and *runx3*, two genes closely related to *runx2*, but involved in the chondrogenic lineage, were expressed (*runx3* expression is shown in Figure 6). The master regulator of chondrogenesis, *sox9*, was highly expressed in the notochord, confirmed by qPCR (Figure 4E). Transcripts of cofactors such as *sox5*, *sox6* that bind to *sox9* in order to activate *col2a1* transcription, were found (Figure 6). In terms of chondrogenic cell differentiation, transcripts of many factors (*sox9*, *sox6*, *sox5*, *ihhb*, *tgfb3*, *col2a1)* were detected (Figure 6). A strong up-regulation of *tgfb1*, confirmed with qPCR, was observed in T3 (Figure 4A).

### Overall activity in the notochord during ontogeny

**Figure 7:**
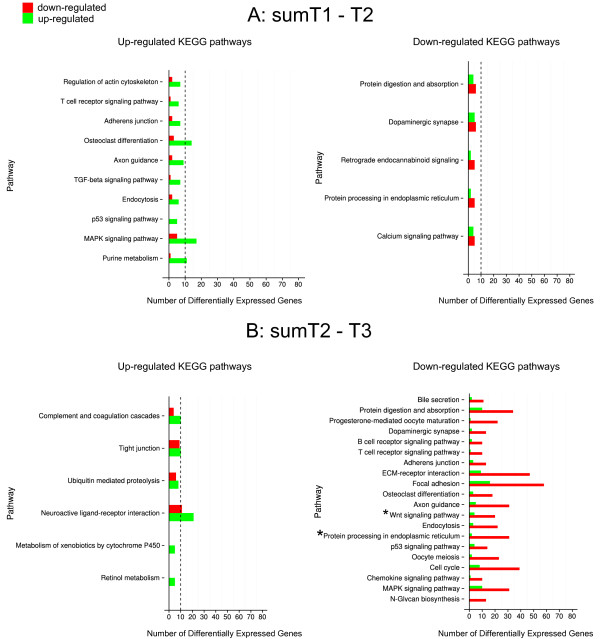
**Actively regulated KEGG pathways in salmon notochord during segmentation.** Numbers of differentially expressed genes (DEGs) were grouped by KEGG ID in pathways. Only genes with a blast score over 300 and fold change >1.5 from NOISeq were counted as DEGs and plotted in KEGG. Up-/down-regulated KEGG pathways from sumT1-T2 (510 ± 20 d°-610 ± 20 d°, pre-segmentation) were listed in **(A)**. Up-/down-regulated KEGG pathways from sumT2-T3 (610 ± 20 d°- 710 ± 20 d°, segmentation) were listed in **(B)**. Up-regulated pathways were ranked by the ratio of the number of up-/down-regulated genes (shown if the ratio is above 1), and vice versa for down-regulated pathways. Pathways with fewer than 10 DEGs (dotted vertical line) were considered to be insufficient for inclusion. Full maps of two pathways (asterisk marked) are illustrated in Additional file 4: Figure S3.

KEGG analysis of differentially expressed pathways between sumT1-T2 (Figure 7A) and sumT2-T3 (Figure 7B) revealed that most pathways were down-regulated between T2 and T3. Considering pathways with above 10 genes being regulated (marked by dotted line in Figure 7), comparisons revealed for up-regulated genes T1-T2: osteoclast differentiation, MAPK signalling pathway and purine metabolism; T2-T3: Neuroactive ligand receptor interaction. The numbers of up-regulated genes were few compared to those down-regulated, while the analysis revealed no pathways for T1-T2, and a comparison of T2 and T3 showed many pathways being down-regulated, including focal adhesion, ECM receptor interaction, cell cycle, protein digestion and absorption, protein processing in the endoplasmic reticulum etc. (Figure 7B). The canonical Wnt pathway was down-regulated from the T2 to T3 stage (Figure 7B), and the full map with DEGs plotted is shown in Additional file 4: Figure S3A. Interestingly, two genes (KEGG ID) repressing the canonical Wnt pathway (*sfrp1* and *dkk2*) and another gene (*smad4*), were up-regulated from T2-T3, and 11 other genes (*wnt2*, *lrp5/6*, *smad4*, *EP300*, *fosl1*) were down-regulated, suggesting a complete picture of down-regulation of the pathway from T2-T3. Another KEGG pathway (protein processing in the endoplasmic reticulum) consisted of 31 down-regulated DEGs and only two up-regulated genes, as shown in Additional file 4: Figure S2B.

### Spatial expression of *col11a2* in the notochord

**Figure 8:**
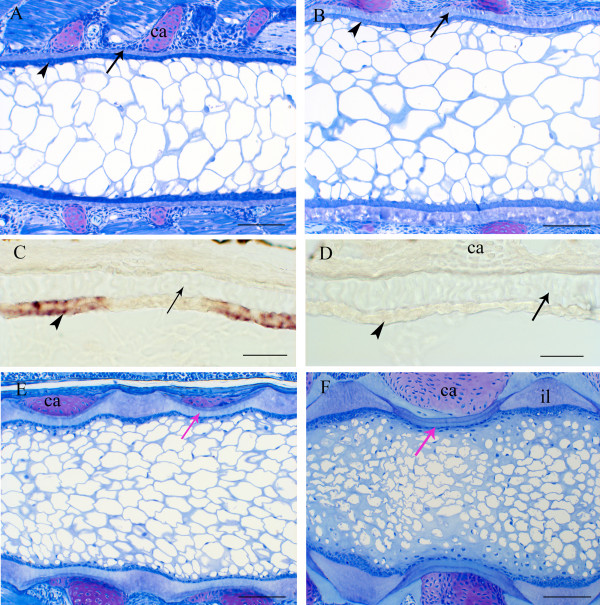
**Notochord morphology and in situ hybridization of *col11a2*. (A, B)** show the morphology before, during and after mineralisation start in the notochord sheath. **(A)** The non-segmented notochord at 500 d°. Arrow: notochord sheath; arrowhead: chordoblasts; ca: cartilaginous arcualia. Scale bar: 100 um. **(B)** Notochord at start of mineralisation in the sheath (arrow), and slightly segmented chordoblasts (arrowhead). Scale bar: 100 um. **(C)** Tissue-specific, segmental gene expression of *col11a2* antisense in chordoblasts (arrowhead) in the notochord at 710 d°. No staining in the notochordal sheath (arrow). Scale bar: 20 μm. **(D)** No background signal was found using the sense probe of *col11a2* (710 d°). Arrowhead: chordoblasts, arrow: notochord sheath. Scale bar: 20 μm. **(E)** Notochord and surroundings, 1000 d°. Red arrow indicates the mineralised chordacentrum, the earliest vertebral element. Ca: arcualial cartilage. **(F)** Differentiated chordacentrum (red arrow), 1300 d°. Notice the thickened notochordal sheath between segments (il: intervertebral ligament), and the thin remaining part of the sheath. Ca: arcualial cartilage. Scale bar: 100 μm.

The notochord in the stages studied is composed of a collagenous sheath, a single layer of chordoblasts, and a central core of vacuolated cells – the chordocytes. At 500 d° (Figure 8A), the notochord is morphologically unsegmented, while at 700 d°, early segmentation becomes visible (Figure 8B). The changes in the transcriptome that occurs between 500 and 700 d° may largely represent a preparation for the profound structural changes that take place, particularly in the sheath when mineralisation commences, ultimately to form the chordacentrae (Figure 8E). The sheath increases substantially in the intervertebral regions (Figure 8E-F), forming a thick intervertebral ligament, while in the region of the chordacentrae, growth in thickness of the sheath ceases (Figure 8F). In situ hybridization was performed on longitudinal cross-sections of notochord at 710 dº and spatial expression of *col11a2* was found segmentally in the chordoblast layer, while no expression was found in the chordocytes (Figure 8C). No background signal was found using the sense probe of the gene (Figure 8D), nor was segmental expression of *col11a2* observed at 510 dº (data not shown). This suggests differentiation of a distinct population of cells that expressed *col11a2* during notochord segmentation. Both RNA-seq and qPCR have confirmed that *col11a1* was down-regulated from T2 to T3, although inconsistent results were obtained between the two methods for *col11a2*.

### Building and mineralisation of the notochord sheath

**Figure 9:**
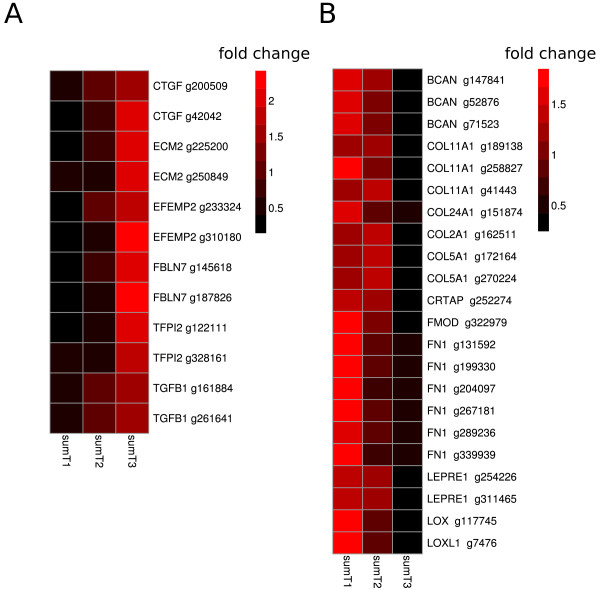
**Differentially expressed ECM genes in the salmon notochord.** Genes related to ECM annotated from Gene Ontology (GO) were merged with differentially expressed gene list output from NOISeq with threshold 0.8 in summed temporal libraries T1-T3 comparison, thereby showing differentially expressed ECM genes from T1 to T3 in **(A,B)**. Heatmap of RNA-seq expression of these genes in SumT1, SumT2 and SumT3 notochord libraries were showed as fold change to the average expression of all summed libraries. Up-regulated ECM genes were listed in **(A)** and down-regulated genes in **(B)**.

To further elucidate which proteins build up the notochordal sheath, extracellular matrix (ECM) genes were retrieved from the annotated RNA-seq pool using Gene Ontology. Only genes with more than 100 reads in the highest coverage of all libraries were further clustered (k-means) over time (T1-T3), in order to find patterns in expression of ECM proteins for the pre-segmented notochord (510–610 dayº), and during segmentation of the notochord (610–710 dayº). K-mean clusters were filtered, and DEGs confirmed by NOISeq were demonstrated in all ECM genes. In genes that were up-regulated from T1 to T3 only six annotations were identified (*ctgf1, acm2, efemp2, fbln7, tfp2 and tgfb1*, Figure 9A). In genes down-regulated from T1 to T3, as many as ten with the same annotation were found; *bcan*, *col11a1*, *col24a*1, *col2a1*, *crtap*, *fmod*, *fn1*, *lepre1*, *lox* and *loxl* (Figure 9B).

## Discussion

About 65000 salmon gene transcripts were expressed in the notochord during the developmental stages analysed. This is an expected number of transcripts, considering the partial tetraploidy of salmonids [29]. In for example zebrafish, which have undergone only one teleost-specific genome duplication in the course of evolution, ~51000 transcripts were identified in a tissue gene expressional study using a similar genome-mapping method [30]. *De novo* assembly of the RNA-seq data could have been implemented, but this method may produce a much higher number of predicted transcripts, as has been observed in rainbow trout (*Oncorhynchus mykiss*) and catfish (*Ictalurus punctatus*), where *de novo* assembly produced about ~130000 and ~370000 predicted transcripts respectively [31,32]. With an average predicted coding sequence length of around 872 bp, the results are comparable to what was predicted using *de novo* assembly methods in catfish [32], but much longer than that obtained in rainbow trout [31]. Of the ~65000 predicted transcripts obtained in the present study, annotation was retrieved for ~55000 transcripts (83%), which is a much higher rate compared to rainbow trout, where only 50% transcripts were annotated [32]. However, due to the fragmented structure of the current salmon genome assembly, which consists of a large Sanger-sequenced data-set with a contig N50 of 9342 bp, it was difficult to predict whole protein lengths and therefore also splice variants in a large fraction of the expressed genes. Moreover, due to the recent genome duplication in salmonids, many paralogous genes display high sequence similarity, which may cause cross-mapping, leading to assembly errors.

Quantitative PCR was used to validate gene expression profiles. Twelve out of thirteen DEGs from NOISeq were confirmed by qPCR, which indicates the high reliability of NOISeq for detecting DEGs. qPCR appeared to be more sensitive than RNA-seq in detecting differentially expressed genes, which was expected due to presence of isoforms which share mapping unequally, a fragmented genome and lack of replicate samples in RNA-seq [33]. Interestingly, there was a high correlation in gene expression data among the hox clusters, as they all displayed collinearity in their expression patterns, in spite of some of them being expressed at a very low level. This gene family consist of very well-characterized full-length genes identified by manual cloning [15]; therefore a fragmented genome might not have affected gene expression profiles of these genes. The lack of replicates also made it difficult to calculate differential gene expression, but by using NOISeq we were able to calculate differential gene expression profiles between stages by combining the expression values of three spatial libraries (A, M, P). NOISeq was chosen mainly because it can simulate technical replicates when few or no replicates are available, which is the case of this study [21]. Since NOISeq does not rely on parametric assumptions, it is also more effective in both identifying differentially expressed transcripts with low read counts and reducing the rate of false discoveries [21].

Of all the 118 salmon hox genes including 8 pseudogenes, 71 were expressed in the developing notochord, and also collinear expression was observed in corresponding hox clusters along the anterior-posterior axis. Interestingly, our study shows that duplicated Hox cluster pairs in salmon have different gene expression patterns, and also this is most obvious in HoxBa clusters (Figure 5A). This fact could be linked slow evolution of HoxBa, both in number of genes and in their gene and intergenic sequences [15]. Our data may therefore suggest that HoxBa have evolved different functional roles in the notochord. So far, only one study in teleosts have previously identified expression of four hox genes in the notochord, among which *hoxb5* displayed the most posterior and highest expression within the notochord [14]. In the notochord, we also found the highest expression in HoxB clusters. Another RNA-seq study of whole eel embryos (*Anguilla Anguilla*), which includes the notochord, has also shown that the HoxBa has the highest expression [34]. Furthermore, clustering of all hox expression patterns in the notochord indicated that highly-expressed hox genes increase in expression at 610 day, and this expression is retained until 710 day (G5 node, in Figure 5C). Interestingly, the expression levels of all hox genes were more similar in the medial part of the notochord throughout the study period than in other regions, which is in accordance with the observation that the initial chordacentrae develop there [9,12,13]. Another point was that several genes found in the A and B clusters show posterior expression patterns. Prince et al. [14] have observed a similar posteriorisation of hox gene expression in the notochord compared to surrounding tissues, including the CNS and paraxial mesoderm. Here, there are two plausible explanations; either hox genes have a more posterior fate in the notochord, or the posterior end of the notochord represents its own patterning unit. In the latter case, we would not be able to detect collinearity in the posterior notochord, since all this tissue was in the posterior sample. But as most of the hox genes display a posteriorisation of expression, it is likely that the latter assumption is to some extent correct.

A wide search for factors implicated in tissue mineralisation resulted in finding of chondroblast-specific “master genes” expressed in notochord such as *sox6*, *sox5* and *sox9*[35], in addition to other genes associated with the fate of chondroblasts, such as *tgfb3, ihhb* and *col2a1*. However, no genes associated with the fate of specific osteoblasts, such as *runx2*, *osterix* and *bgp*, were found. The chondrogenic genes *Sox5* and *Sox6* have previously been shown to be expressed in the zebrafish notochord. Knock-out of both genes in zebrafish inhibits ECM formation in the notochord, which subsequently induces apoptosis in notochord cells [36]. Interestingly, the *sox9a/b* mutant in zebrafish displays a truncated notochord, supporting the notion of a function of *sox9* in chordocytes [37]. *Ihhb* is essential for chondrogenesis in vertebrates, including teleost fish, where its expression has been associated with chondrogenic tissue [38]. In the same study, *ihhb* was identified in the notochord, further supporting the concept of evolutionary closeness between chondroblasts and chordoblasts. *Tgfb3* was another gene found to be expressed in the notochord, and this is also expressed in a number of cartilage tissues in zebrafish [39]. *Tgfb3* is also highly expressed in the notochord during early development in the zebrafish, but as development progresses it ceases to be expressed in the notochord. Similarly, we observed a reduced expression of *tgfb3* over time in salmon notochord. One of the most highly expressed genes within the notochord is the major collagen of hyaline cartilage, *col2a1*[40]. This collagen type displays a much higher level of expression than the major type I collagen (*colI*) found in bone, further confirming the chondroblast-like nature of chordoblasts [13]. Since the notochord appeared early in chordate evolution, as a key structure characterising the clade, and anticipating by far the emergence of hyaline cartilage, which probably first appeared in jawed fish, it may be fair to say that chondroblasts are chordoblast-like, having co-opted morphogenic pathways that evolved together with the notochord in the pre-chordate lineage, early in the Cambrian (see discussion in [12]).

Both qPCR and RNA-seq employed in this study show a significant down-regulation of the fibril collagen *col11a1* at onset of mineralisation (610dº -710dº), suggesting an overall reduced synthesis and re-modelling of extracellular matrix in the notochord sheath concurrent with segmentation and mineral crystal deposition. Nine other ECM genes were also down-regulated at onset of mineralisation (610dº -710dº); these included *bcan*, *col24a*1, *col2a1*, *crtap*, *fmod*, *fn1*, *lepre1*, *lox* and *loxl*. It was also shown that at 710dº, *col11a2* was spatially expressed in regions not undergoing mineralisation – the sites of the prospective intervertebral joints – which suggests that the overall down-regulation of genes may be due to compartmentalisation of expression (Figure 8C). Likewise, expression of ALP in chordoblasts in segments where mineralisation occurs substantiates such a notion. In the notochord, it has been observed that induction of ECM-specific genes over time (*ctgf*, *efemp2* or *fibulin 4*, *fbln7* and *tfpi2*), and proteins encoded by these genes, might further contribute to the specificity of the segmentation and mineralisation processes. In the KEGG analysis of differentially expressed genes performed in the present study, we discovered a massive down-regulation of many pathways between 510–710 dayº. However, only the Wnt pathway was found to be down-regulated early, in the period between 610 and 710 day º, which may indicate that the Wnt pathway is involved in the segmentation and subsequent mineralisation of the chordacentrae (Figure 7B). Indeed, key Wnt signaling pathway genes were affected, including *fzd2*, *fosl*, *nr1c2*, *wnt2*, *lrp5/6*, *fosl*, *sfrp1* and *dkk2* (Additional file 4: Figure S2A).

Experimental manipulations in the chick embryo have excluded the possibility that the notochord plays a role in segmentation of the vertebral column, indicating that subdivision of the sclerotome alone leads to this patterning in avian species, and probably in all amniotes [41]. A study performed on the Japanese medaka (*Oryzias latipes*), supports the notion that this mechanism is also involved in teleost species [42]. Prior to mineralisation of the notochord sheath in medaka, sclerotome-derived cells, which express *col10a1*, migrate to surround the notochord at segments where the prospective chordacentrae are to develop. The cells differentiate into osteoblasts, and it has been suggested that they, rather than the notochord epithelium, secrete molecules that induce mineralisation of the sheath, thus forming the chordacentrae and subsequently depositing osteoid to form perichordal vertebral bone. Such a mechanism, in which segmentation is independent of the notochord, would be similar to that described in amniotes [41]. On the other hand, in zebrafish, sclerotomal cells do not migrate to surround the notochord in strictly segmented compartments [43], and, in salmon, the notochord epithelium expresses ALP – a key enzyme in tissue mineralisation – in a spatio-temporal manner that coincides with development of the chordacentrae; here osteoblast precursor cells surrounding the notochord do not express ALP, and thus do not appear to play a role in the mineralisation of the notochord sheath. In medaka, an alternative mechanism for the accumulation of osteoblast precursors at specific sites along the notochord [42] may be that the cells respond to unidentified chemoattractant guiding factors that are segmentaly secreted by the notochord epithelium. Indeed, to ensure an uninterrupted development of the vertebrae, these cells need to be in place before the chordacentrae form. Moreover, in zebrafish, when the osteoblasts surrounding the notochord are abolished, the initial development of chordacentrae proceeds normally, but they continue to grow, so that the spaces separating adjacent centrae become reduced, in some cases finally leading to partial fusion [8]. Hence, osteoblasts that are to form perichordal bone may provide signalling factors that inhibit the notochord epithelium in its secretion of molecules that promote mineralisation of the sheath, arresting further growth of the chordacentrae, and, by extension, defining a boundry with non-mineralised segments that correspond to where prospective intervertebral ligaments are to develop. Here, we have identified a metameric expression of *col11a2* within the notochord that colocalizes with these segments. Apart from being an extracellular structural matrix protein found in the core of the collagen type II fibril, collagen type XI may inhibit maturation of osteoblasts [44] in the region where intervertebral ligaments develop – here, instead of osteoblasts, fibroblasts condense around the notochord to form collagenous tissue [11]. One of the end players in the Wnt pathway, which is down-regulated at the onset of segmentation, is collagen type XI. This molecule may act as a downstream inhibitor of mineralisation along the whole notochord prior to segmentation, as expression of *col11a2* is, at the onset of segmentation, confined to regions where mineralisation does not occur. Hence, through controlling inhibitory signals, the Wnt pathway may play a key role in notochord segmentation.

The gathering of osteoblast precursors around the notochord, and the inhibitory effect of osteoblasts on growth of the chordacentra, suggests a reciprocal interaction between notochord and surrounding tissue. However, our finding of a complex dynamic regulation of a range of anterior-posterior polarity genes and the segmental expression of *col11a2* within the notochord epithelium supports the notion that autonomous segmental patterning processes exist in the teleost notochord.

## Conclusions

Sequencing and partial analysis of the notochord transcriptome of salmon, the first dataset of its kind from a vertebrate, has provided new insight into the cellular and molecular genetic mechanisms of this organ. The collinear expressions of 18 hox clusters in the notochord represented by 71 hox genes, suggest a possible role of hox genes in anterior-posterior patterning of the notochord. When notochord segmentation occurs, we find an extensive down-regulation of genes, which may reflect a shutdown of complex regulatory developmental pathways leading up to this event. Expression of *col11a2* was detected in segments where prospective intervertebral ligaments develop, and we hypothesise that the Wnt pathway, where collagen type XI is one of the end players, may play a key role in segmental patterning. Furthermore, the expression of genes that determine differentiation of chondroblasts indicates that cartilage may have evolved after the co-option of morphogenic pathways that initially evolved together with the notochord in the pre-chordate lineage.

## Competing interests

The authors declare that they have no competing interests.

## Authors’ contributions

SG, AW, GT and HK initiated the project. HK, SW and CK dissected and sampled all notochords. SW performed all the molecular analysis with guidance from AW. Bioinformatical analysis was performed by TF in close collaboration with SW and AW. CK performed in situ hybridization analysis. Histological sections were provided by HK. SW prepared most of the figures and tables in the manuscript. The manuscript was drafted mainly by SW, HK, CK, SG, GT and AW, with contributions from TF. All authors read and approved the final manuscript.

## Supplementary Material

Additional file 1: Table S1 RNA library and reads mapping summary. Quality of RNA libraries and reads, as well as mapping statistics summarised in one table. Click here for file

Additional file 2: Figure S1 Quality check summary. **(A)** Quality score of nucleotide positions in short reads from a titration run. **(B)** Nucleotide distribution in short reads from a titration run. Click here for file

Additional file 3: Table S2 Primer sequence and additional information of selected genes in qPCR validation and in situ hybridization. Detailed information about 23 selected genes used in qPCR validation of RNA-seq gene expression. One-way ANOVA was used to define significance of differentially expressed gene (DEG) if p<0.05 among summed libraries (both spatially and temporally). DEG analysis using NOISeq was used to detect genes from RNA-seq dataset. Click here for file

Additional file 4: Figure S2 Full KEGG pathway maps for two important pathways. Wnt signaling pathway and protein processing in endoplasmic reticulum are illustrated in **(A)** and **(B)**, respectively. Click here for file
