# Supplementary figures and images for: Transcriptome sequencing of Atlantic salmon (Salmo salar L.) notochord prior to development of the vertebrae provides clues to regulation of positional fate, chordoblast lineage and mineralisation
Source: BMC Genomics. 2014 Feb 19;15:141. doi: 10.1186/1471-2164-15-141 (PMC3943441; doi:10.1186/1471-2164-15-141)

A

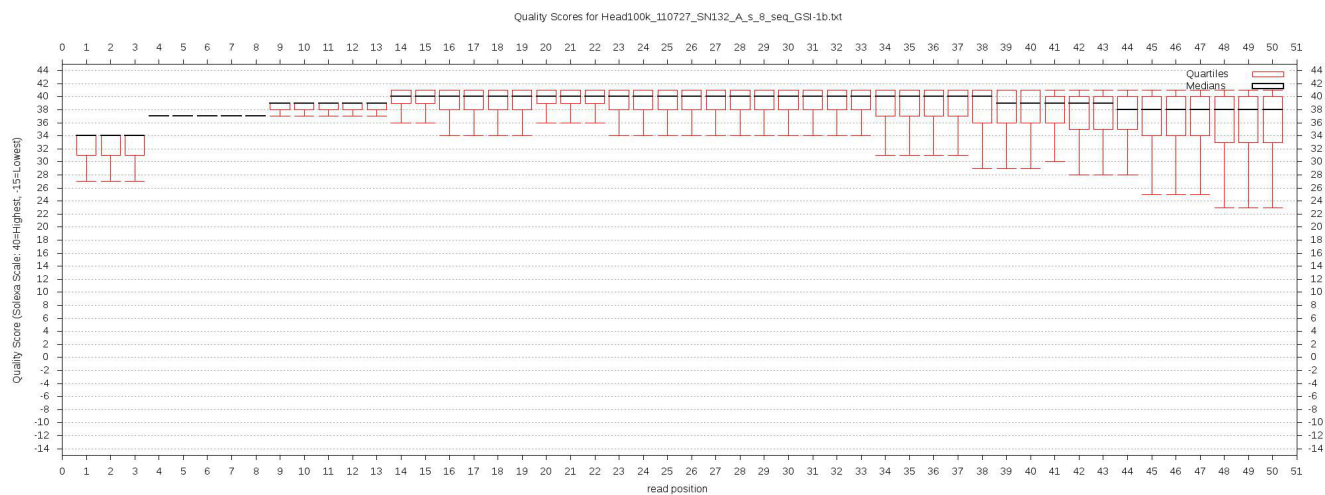

B

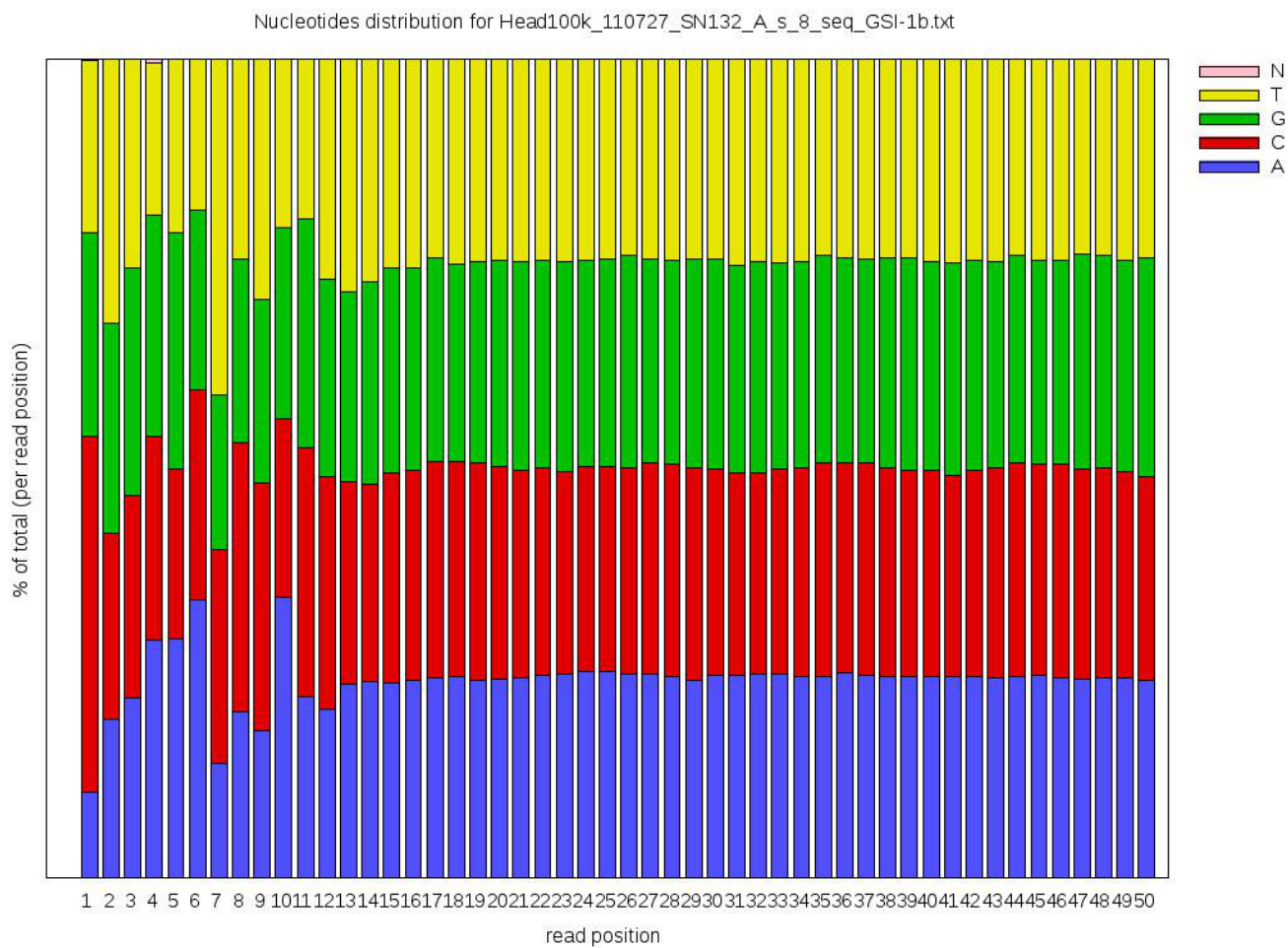

Supplement: Additional file 2: Figure S1 — Quality check summary. (A) Quality score of nucleotide positions in short reads from a titration run. (B) Nucleotide distribution in short reads from a titration run. [file 1471-2164-15-141-S2.pdf]
