# Supplementary material for: Transcriptome sequencing of Atlantic salmon (Salmo salar L.) notochord prior to development of the vertebrae provides clues to regulation of positional fate, chordoblast lineage and mineralisation
Source: BMC Genomics. 2014 Feb 19;15:141. doi: 10.1186/1471-2164-15-141 (PMC3943441; doi:10.1186/1471-2164-15-141)
Supplement: Additional file 4: Figure S2 — Full KEGG pathway maps for two important pathways. Wnt signaling pathway and protein processing in endoplasmic reticulum are illustrated in (A) and (B), respectively. [file 1471-2164-15-141-S4.pdf]

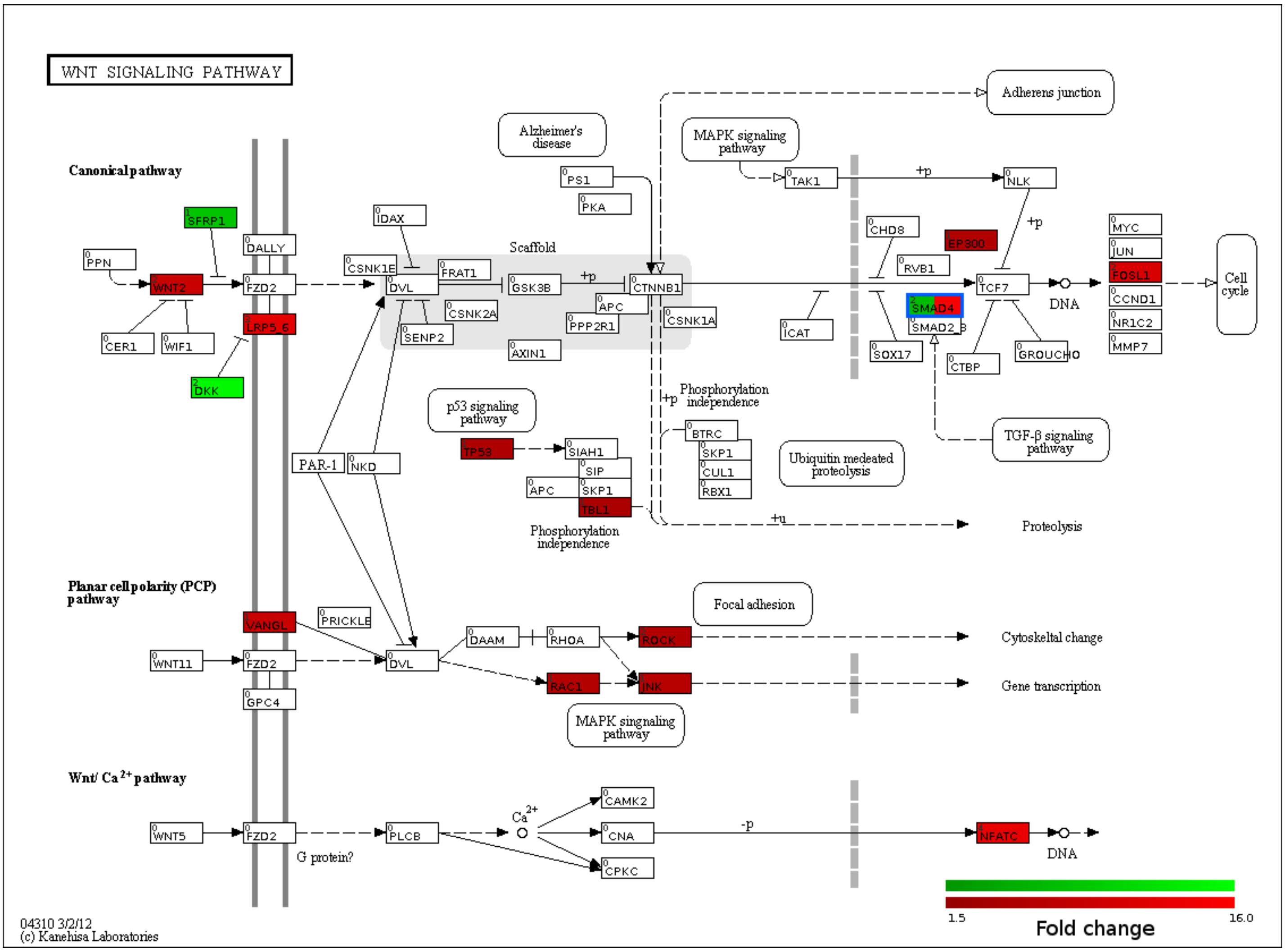
WNT SIGNALING PATHWAY
Canonical pathway
SFRP1
DALLY
PPN
WNT2
FZD2
LRP5_6
CER1
WIF1
DKK
IDAX
CSNK1E
DVL
FRAT1
GSK3B
CSNK2A
SENP2
APC
PPP2R1
AXIN1
CTNNB1
CSNK1A
Scaffold
Alzheimer's disease
PS1
PKA
MAPK signaling pathway
TAK1
NLK
Adherens junction
CHD8
EP300
RVB1
SMAD4
SMAD2_3
ICAT
SOX17
CTBP
GROUCHO
TCF7
DNA
MYC
JUN
FOSL1
CCND1
NR1C2
MMP7
Cell cycle
TGF-β signaling pathway
p53 signaling pathway
TP53
SIAH1
SIP
SKP1
APC
TBL1
Phosphorylation independence
BTRC
SKP1
CUL1
RBX1
Ubiquitin mediated proteolysis
Proteolysis
PAR-1
NKD
Planar cell polarity (PCP) pathway
VANGL
PRICKLE
WNT11
FZD2
GPC4
DVL
DAAM
RHOA
ROCK
RAC1
JNK
Focal adhesion
Cytoskeltal change
Gene transcription
MAPK singnaling pathway
Wnt/ Ca2+ pathway
WNT5
FZD2
G protein?
PLCB
Ca2+
CAMK2
CNA
CPKC
NFATC
DNA
1.5
16.0
Fold change
04310 3/2/12
(c) Kanehisa Laboratories

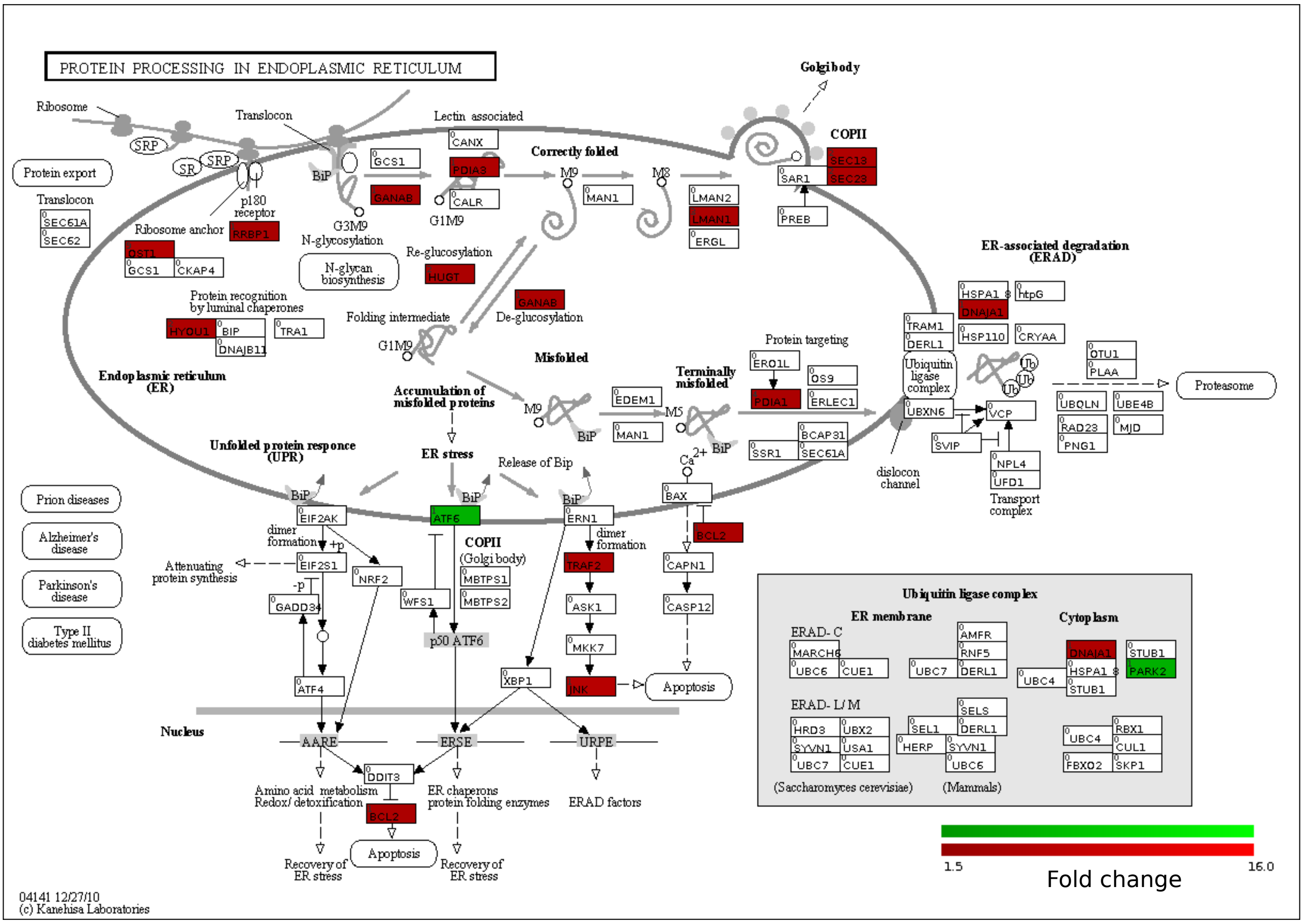
PROTEIN PROCESSING IN ENDOPLASMIC RETICULUM
Ribosome
SRP
SR
Translocon
Protein export
Translocon
SEC61A
SEC62
p180 receptor
Ribosome anchor
RRBP1
OST1
GCS1
CKAP4
BiP
G3M9
N-glycosylation
GCS1
Lectin associated
CANX
PDIA3
GANAB
CALR
G1M9
Correctly folded
M9
MAN1
M8
LMAN2
LMAN1
ERGL
Golgi body
COPII
SEC13
SEC23
SAR1
PREB
N-glycan biosynthesis
Re-glucosylation
HUGT
GANAB
De-glucosylation
Folding intermediate
Protein recognition by luminal chaperones
HYOU1
BIP
DNAJB11
TRA1
G1M9
Endoplasmic reticulum (ER)
Misfolded
Accumulation of misfolded proteins
EDEM1
M9
M5
MAN1
BiP
Terminally misfolded
ERO1L
PDIA1
OS9
ERLEC1
SSR1
BCAP31
SEC61A
Protein targeting
ER-associated degradation (ERAD)
HSPA1
DNAJA1
htpG
TRAM1
DERL1
HSP110
CRYAA
Ubiquitin ligase complex
UBXN6
VCP
SVIP
NPL4
UFD1
Transport complex
dislocon channel
OTU1
PLAA
UBQLN
UBE4B
RAD23
MJD
PNG1
Proteasome
Unfolded protein responce (UPR)
ER stress
Release of Bip
Ca2+
BiP
BAX
BCL2
CAPN1
CASP12
Apoptosis
Prion diseases
Alzheimer's disease
Parkinson's disease
Type II diabetes mellitus
EIF2AK
dimer formation
+p
EIF2S1
Attenuating protein synthesis
-p
GADD34
NRF2
ATF4
ATF6
WFS1
COPII (Golgi body)
MBTPS1
MBTPS2
p50 ATF6
ERN1
dimer formation
TRAF2
ASK1
MKK7
JNK
XBP1
Apoptosis
Nucleus
AARE
ERSE
UPRE
DDIT3
Amino acid metabolism Redox/ detoxification
BCL2
Apoptosis
ER chaperons protein folding enzymes
ERAD factors
Recovery of ER stress
Recovery of ER stress
Ubiquitin ligase complex
ER membrane
Cytoplasm
ERAD- C
MARCH6
UBC6
CUE1
AMFR
RNF5
UBC7
DERL1
DNAJA1
HSPA1
STUB1
PARK2
UBC4
STUB1
ERAD- L/ M
HRD3
UBX2
SYVN1
USA1
UBC7
CUE1
SELS
SEL1
DERL1
HERP
SYVN1
UBC6
UBC4
RBX1
CUL1
FBXO2
SKP1
(Saccharomyces cerevisiae)
(Mammals)
1.5
16.0
Fold change
04141 12/27/10
(c) Kanehisa Laboratories
